# Patterns of regional recurrence in papillary thyroid cancer patients with lateral neck metastases undergoing neck dissection

**DOI:** 10.1186/s40463-017-0221-3

**Published:** 2017-05-31

**Authors:** Jason J. Xu, Eugene Yu, Caitlin McMullen, Jesse Pasternak, Jim Brierley, Richard Tsang, Han Zhang, Antoine Eskander, Lorne Rotstein, Anna M. Sawka, Ralph Gilbert, Jonathan Irish, Patrick Gullane, Dale Brown, John R. de Almeida, David P. Goldstein

**Affiliations:** 1 Department of Otolaryngology—Head and Neck Surgery, University Health Network, University of Toronto, Toronto, Ontario Canada; 2 Department of Medical Imaging, University Health Network, University of Toronto, Toronto, Ontario Canada; 3 Department of Surgery, Division of General Surgery, University Health Network, University of Toronto, Toronto, Ontario Canada; 4 Department of Radiation Oncology, University Health Network, University of Toronto, Toronto, Ontario Canada; 5 Department of Medicine, Division of Endocrinology, University Health Network University of Toronto, Toronto, Ontario Canada

**Keywords:** Papillary thyroid carcinoma, Neck dissection, Regional recurrence

## Abstract

**Background:**

Practice variability exists for the extent of neck dissection undertaken for papillary thyroid carcinoma (PTC) metastatic to the lateral neck nodes, with disagreement over routine level V dissection.

**Methods:**

We performed a retrospective medical record review of PTC patients with lateral neck nodal metastases treated at University Health Network from 2000 to 2012. Predictive factors for regional neck recurrence, including extent of initial neck dissection, were analyzed using Cox regression.

**Results:**

Out of 204 neck dissections in 178 patients, 110 (54%) underwent selective and 94 (46%) had comprehensive dissection including level Vb. Mean follow-up was 6.3 years (SD). Significant predictors of regional failure were the total number of suspicious nodes on preoperative imaging (*p* = 0.029), largest positive node on initial neck dissection (*p* < 0.01), and whether patients received adjuvant radiotherapy (*p* = 0.028). The 5-year ipsilateral regional recurrence rate was 8 and 9% with selective and comprehensive dissection, respectively (*p* = 0.89).

**Conclusion:**

The extent of neck dissection did not predict the probability of regional recurrence in PTC patients presenting with lateral neck metastases.

## Background

There is no clear consensus regarding the extent of lateral neck dissection required in the treatment of papillary thyroid carcinoma (PTC). For patients presenting with clinical, radiographic or cytologic evidence of lateral lymph node metastases, the standard of care includes lateral neck lymph node dissection [1, 2]. However, variability in clinical practice persists regarding the levels of dissection required. Some surgeons perform a comprehensive neck dissection – including a formal level Vb dissection – with the intention of potentially lowering the rate of regional recurrence [3–5]. Others, believing that elective level Vb dissection is unwarranted and results in greater morbidity [6], argue that a formal level Vb dissection should only be performed given sufficient clinical and radiographic suspicion of disease in that level [7, 8].

A meta-analysis examining patterns of nodal metastases in patients with PTC and lateral neck metastases reported level V metastatic disease in 25.3% of cases, with Va and Vb nodal positivity in 7.9 and 21.5% of patients, respectively [3]. Based on this high rate of Vb involvement, the authors recommended a comprehensive lateral neck dissection including levels IIa, IIb, III, IV and Vb in all patients with PTC and lateral neck disease. However, the data from this meta-analysis for level Vb recurrence was pooled from only 3 uncontrolled case series with a small sample size (pooled *n* = 137). The general approach to the management of the neck for PTC at the University Health Network (Princess Margaret Cancer Center and Toronto General Hospital) has been to base the levels of neck dissection on the extent of disease as determined with preoperative ultrasound and/or cross-sectional CT imaging. Given the high reported rate of nodal metastases on pathologic examination in level Vb we sought to determine the rate and patterns of regional failure in patients undergoing neck dissection for thyroid cancer at our institution.

## Methods

### Study design

We conducted a retrospective review of all consecutive PTC patients with lateral neck metastases treated at the University Health Network (UHN) from January 1, 2000 to August 1, 2012. We obtained approval from the UHN Research Ethics Board. Subjects were identified by screening all patients with both a diagnosis of thyroid cancer and any billing code for lateral neck dissection. Collection of data from the charts went up to March 31, 2016. Adult patients (>18 years of age) who underwent unilateral or bilateral lateral neck dissection for regional metastases from PTC were eligible for inclusion. Neck dissections could have been performed either concurrently or no more than 5 years after the initial thyroidectomy. We excluded patients if they had any pathology other than PTC (including insular cell carcinoma or Hurthle cell carcinoma), a history of previous neck dissection, no metastases identified in the neck dissection specimen on histopathology, received ≥2 radioactive iodine (RAI) treatments prior to neck dissection, incomplete operative notes where the extent or type of neck dissection could not be determined, or if they were lost to follow up within the first 12 months after surgery.

The general approach to neck dissection for PTC at the University Health Network has been to perform a resection of the levels of the neck with radiographic or clinical suspicion of metastases; however, the extent of dissection was determined ultimately at the discretion of the treating surgeon. General indications for performing a comprehensive dissection of level Vb in the absence of radiographic evidence of metastases included bulky nodal metastases and/or large nodal disease, significant level IV metastases or surgeon preference.

We extracted data on patient demographics, the extent of neck dissection performed, pathology results including nodal ratio of dissection specimens, adjuvant treatment given and development of regional recurrence. A staff radiologist reviewed all pre- and post-operative computerized tomography (CT) images and collected data on the location, size and total number of nodes suspicious for PTC metastases. Nodes were deemed suspicious if any of the following features were present: cystic and enhancing with small foci of calcifications, enhancing internal solid components and necrotic and enhancing. The size requirement of suspicious lymph nodes were 1.5 cm for level 1B and jugulo-diagastric nodes and 1 cm for all other lymph nodes, taken in context with the aforementioned suspicious features.

Two study authors independently reviewed operative notes to determine the extent of neck dissection performed by level. Disagreements were reconciled through consensus or with the surgeon who performed the case. Patients were separated into two cohorts depending on the extent of the neck dissection they received. The selective dissection group was defined as those that received a neck dissection comprised of levels IIa (+/−IIb), III, IV and often the anterior aspect of level Vb. The comprehensive neck dissection group received formal IIa (+/−IIb) to Vb dissection, which included dissecting the posterior accessory nerve to the anterior border of trapezius and resecting all the nodal tissue below.

### Outcomes and statistics

Our primary outcome was regional recurrence of PTC in the ipsilateral lateral neck. For patients with bilateral neck disease, we analyzed each side separately. Patients were recorded as having a regional recurrence if they had histologically proven PTC in a lymph node on fine needle aspiration (FNA) or salvage neck dissection, or had CT and ultrasound findings consistent with nodal recurrence (based on the above mentioned criteria) with or without elevated or rising thyroglobulin levels. We only classified patients as regional recurrence if they recurred in the lateral neck, and thus we did not count patients with isolated thyroid bed or central compartment recurrences for the purposes of this study. The location of neck recurrence was determined based on imaging.

Comparison of clinical features between selective and comprehensive neck dissection was performed using the Chi-squared test or Fisher’s exact test for categorical variables and Student’s T-test or Wilcoxon rank sum test for continuous variables. Time to neck recurrence was analyzed using the Kaplan-Meier method. Univariate and multivariate analysis was conducted using Cox proportional hazards regression model. Statistical significance was defined as *p* < 0.05. Statistical analysis was performed using SAS version 9.4 and R 3.1.2.

## Results

### Baseline comparison between selective and comprehensive neck dissection groups

After review, 178 patients who underwent 204 neck dissections met the inclusion criteria. Of note, we excluded 11 potentially eligible patients due to incomplete operative notes,16 due to being lost to follow-up, 19 because they had two or more prior RAI treatments, and 14 who presented >5 years with neck disease after their initial thyroidectomy. Of the 204 neck dissections that met inclusion criteria, 110 (54%) were selective and 94 (46%) were comprehensive dissections. There were 26 patients who underwent bilateral neck dissections. Concurrent total thyroidectomy was performed in 169 cases (83%), concurrent completion thyroidectomy was performed in 6 cases (3%), and 29 cases had prior thyroidectomy (14%). The mean age was 44.8 years (SD = 14.9) with 45% (*n* = 91) of patients being older than 45 years of age. The majority of patients (60%, *n* = 123) were female. There were no significant differences between the selective neck dissection patient group and the comprehensive neck dissection group in terms of patient demographics.

On pre-operative staging, 4% (*n* = 4) of patients undergoing selective neck dissection and 19% (*n* = 18) patients undergoing a comprehensive neck dissection had radiographic evidence of level V disease (*p* < 0.001). Patients undergoing a comprehensive neck dissection had a greater mean number of radiographic suspicious nodes (3.6 vs 2.6, *p* = 0.034) and greater diameter in the largest node (2.4 cm vs. 1.6 cm, *p* < 0.01) compared with those who underwent selective neck dissection. On pathologic assessment of neck dissection specimens (Table 1), the comprehensive dissection group had a greater mean number of positive nodes (6.7 vs. 5.2, *p* =0.03) and greater number of total nodes removed (34.8 vs. 27.8, *p* < 0.01) compared with the selective neck dissection group, but no significant difference in whether there were nodes with extracapsular extension, nodal ratio or mean diameter of the largest node.

**Table 1:** Neck dissection specimen pathology

	Full Sample (*n* = 204)	Comprehensive (*n* = 94)	Selective (*n* = 110)	*P*-value
Extracapsular Extension (ECE)	49 (24%)	22 (23%)	27 (25%)	0.87
Positive Nodes^a^	5.9 (4.8)	6.7 (5.5)	5.2 (4.1)	**0.03**
Total Nodes^a^	31 (17.3)	34.8 (18)	27.8 (16)	**0.004**
Nodal Ratio	21.6%	21.1%	21.9%	0.74
Nodal Size (cm)^a^	2.5 (1.2)	2.7 (1.3)	2.4 (1.1)	0.12

^a^Mean (SD)

Significant *p* values are captured in bold

In terms of adjuvant treatment, almost all patients received adjuvant radioactive iodine. There were no differences between the neck dissection groups in terms of RAI dose, number of treatments and whether external beam radiotherapy was received (Table 2).

**Table 2:** Adjuvant treatment

	Full Sample (*n* = 204)	Comprehensive (*n* = 94)	Selective (*n* = 110)	*P*-value
Received RAI	203 (100)	94 (100)	109 (99)	0.99
RAI Dose^a^	135 (42)	132 (38.2)	138 (45.1)	0.39
Multiple RAI Doses	8 (4)	2 (2)	6 (5)	0.29
Received EBRT	13 (6)	7 (7)	6 (5)	0.58

^a^Mean (SD)

### Outcomes

The mean length of follow up was 75.6 months (SD = 33.7). Mean follow up for the selective and comprehensive groups were 67 and 86 months, respectively. There were a total of 20 regional recurrences in the overall cohort, with 12 in the selective neck dissection group and 8 in the comprehensive neck dissection group. In terms of regional recurrences, 14 were based on pathologic assessment (13 salvage neck dissection pathology and 1 FNA biopsy) and 6 were based on imaging alone without pathologic assessment. Of the latter group, 5 had increasing thyroglobulin levels in addition to suspicious imaging features, while 1 had positive anti-thyroglobulin antibodies.

**Fig. 1:**
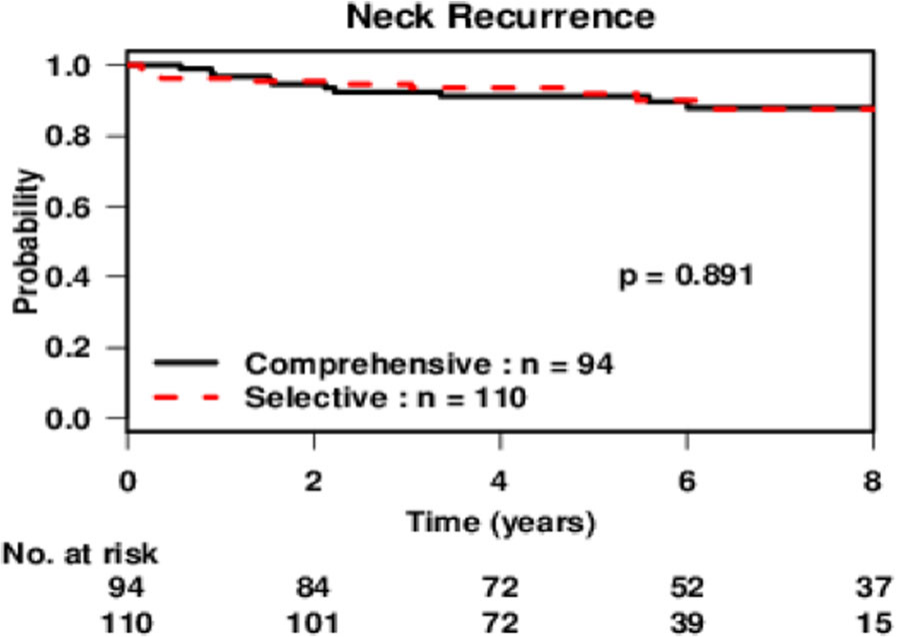
Recurrence-free probability of selective and comprehensive neck dissection over time

The 5-year ipsilateral regional control rate for the entire cohort was 92% (95% CI: 88–96%). The results of the univariate analysis are listed in Table 3. The significant predictors of regional failure were the total number of suspicious nodes on preoperative imaging (*p* = 0.029), largest positive node on initial neck dissection (*p* < 0.01), and whether patients received external beam radiotherapy (EBRT, *p* = 0.028). The type of neck dissection was not predictive of regional recurrence (Fig. 1). The 5-year regional control rate was 91% (86–97) for the selective dissection group and 92% (87–98) for the comprehensive dissection group (*p* = 0.89). Multivariate Cox regression model adjusted for significant factors identified on univariate analysis found that the hazard ratio of selective neck dissection for neck recurrence was 2.55 (95% CI: 0.63–10.38, *p* = 0.19). Multivariate analysis was performed excluding patients who received EBRT and found that selective neck dissection was still not significantly associated with regional recurrence (*p* = 0.26, Table 4).

**Table 3:** Univariate analysis–Cox proportional hazards regression for regional recurrence

Variable	HR (95%CI)	Global *p*-value
Selective Neck Dissection (Ref: Comprehensive)	0.94 (0.39,2.27)	0.89
Age ≥ 45 (Ref: <45)	0.93 (0.39,2.26)	0.88
Concurrent Thyroidectomy (Ref:No)	0.66 (0.21,2.06)	0.48
Extracapsular Extension	1.89 (0.75,4.74)	0.17
Level V Disease (Ref:No)	1.91 (0.64,5.72)	0.25
Total Nodes (CT)	1.26 (1.02,1.55)	**0.029**
Largest Node (CT)	1.24 (0.81,1.89)	0.32
Total Nodes (Path)	1.04 (0.97,1.11)	0.29
Largest Node (Path)	1.56 (1.19,2.05)	**0.0015**
Nodal Ratio (Path)	5.11 (0.38,68.66)	0.22
RAI (Ref: No)	3296373.77 (0,Inf)	1
RAI Dose	1 (0.99,1.01)	0.66
Multiple RAI (Ref: No)	0 (0,Inf)	1
EBRT (Ref: No)	3.45 (1.14,10.37)	**0.028**
Male (Ref: Female)	0.93 (0.38,2.27)	0.87
Age	1 (0.97,1.03)	0.91
Follow-Up (months)	1 (0.98,1.01)	0.61

Significant *p* values are captured in bold

**Table 4:** Multivariate analysis–Cox PH regression model adjusted for total nodes on pre-operative CT and largest node on pathology

Variable	HR (95%CI)	Global *p*-value
Selective Neck Dissection (Ref: Comprehensive)	2.35 (0.53,10.41)	0.26
Total Nodes (CT)	1.29 (1.01,1.65)	**0.039**
Largest Node (Path)	2.06 (1.17,3.6)	**0.012**

Significant *p* values are captured in bold

**Fig. 2:**
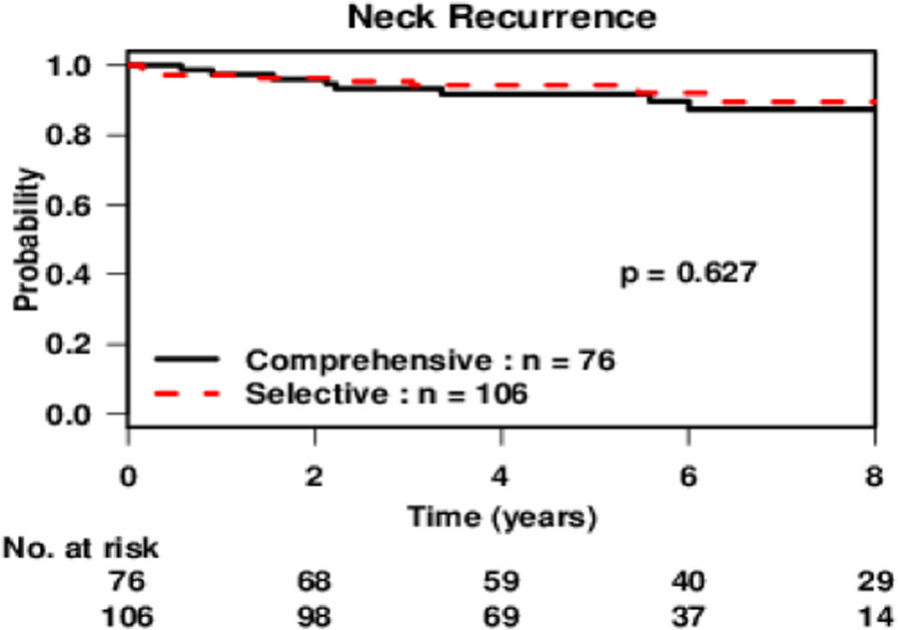
Recurrence-free probability of patients without level V disease on preoperative imaging

A subgroup analysis was performed for those patients who did not have positive level V disease on pre-operative imaging. There were 106 and 76 cases in the selective and comprehensive groups, respectively. Similarly, the type of neck dissection was not predictive of regional recurrence (Fig. 2), with a 5-year regional control rate of 94% (95% CI: 90–99) and 92% (95% CI: 86–98) for the selective and comprehensive groups, respectively (*p* = 0.63).

The location of regional recurrence by type of neck dissection is listed in Table 5. In the selective group, 6 of 12 cases of recurrence (50%) were considered “out-of-neck dissection field” failures, with 5 cases involving level V and 1 case involving in level IIb. For the comprehensive group, 3 of 8 cases of recurrence (37.5%) contained “out-of-field” failures, all in level IIb. The comprehensive and selective groups had no significant difference in the rate of level Vb recurrence (2% vs 3%, *p* = 1.00) or recurrence at any other level.

**Table 5:** Location of regional recurrence by neck dissecton type

	Full Sample (*n* = 204)	Comprehensive (*n* = 94)	Selective (*n* = 110)	*p*
Level IIa Disease	9 (4%)	2 (2%)	7 (6%)	0.18
Level IIb Disease	6 (3%)	5 (5%)	1 (1%)	0.097
Level III Disease	5 (2%)	3 (3%)	2 (2%)	0.66
Level IV Disease	8 (4%)	3 (3%)	5 (5%)	0.73
Level Va Disease	2 (1%)	1 (1%)	1 (1%)	1.00
Level Vb Disease	5 (2%)	2 (2%)	3 (3%)	1.00
Total Nodes^a^	2.5 (2.8)	2.8 (3.6)	2.2 (1.6)	0.73
Largest Node^a^	1.5 (0.7)	1.4 (0.5)	1.7 (1)	0.47

^a^Mean (SD)

All five patients in the selective dissection group with recurrence in level V did not have any suspicious level V adenopathy on their pre-operative CT. These patients had on average 7 positive lymph nodes on their initial neck dissection specimen, with an average nodal ratio of 0.21. The largest positive node for these patients is on average 2.5 cm. The salvage neck dissection pathology showed fewer positive nodes for the comprehensive group (1.7 vs 4, *p* = 0.038) and more total nodes removed for the selective group (13.2 vs 6.2, *p* = 0.039), but no difference in nodal ratio or size of the largest positive node (Table 6).

**Table 6:** Revision neck dissection specimen pathology

	Full Sample (*n* = 204)	Comprehensive (*n* = 94)	Selective (*n* = 110)	*p*
Positive Nodes^a^	2.7 (2.6)	1.7 (1.6)	4 (3.1)	**0.038**
Total Nodes^a^	9.2 (7.8)	6.2 (6.5)	13.2 (7.9)	**0.039**
Nodal Ratio	31.8%	41.9%	24.6%	0.35
Largest Node (cm)^a^	1.7 (0.9)	1.6 (0.5)	1.9 (1.1)	0.44

^a^Mean (SD)

Significant *p* values are captured in bold

## Discussion

The 2012 American Thyroid Association (ATA) consensus statement on lateral neck dissection for PTC states that “lateral neck dissection performed for macroscopic DTC metastases should be the selective neck dissection of levels IIa, III, IV and Vb.” [1] However, while the more updated 2015 ATA guideline strongly recommends that “therapeutic lateral neck compartmental lymph node dissection should be performed for patients with biopsy-proven metastatic lateral cervical lymphadenopathy,” the extent of surgery or which nodal compartments to dissect are no longer specified [2]. The approach to nodal management at the University Health Network, Princess Margaret Cancer Center generally has been to base the extent of neck dissection on the location and volume of disease seen on pre-operative imaging, and to avoid the comprehensive level Vb neck dissection where feasible in order to reduce potential morbidity. This conflicts with several reports in the literature, which argue that a comprehensive level Vb dissection is always necessary given a high rate of level Vb metastases ranging from 15 to 40%, with Eskander et. al. in their meta-analysis of 18 pooled studies reporting level Vb disease in 21.5% of patients [2]. Other authors since the meta-analysis have also argued for routine level V comprehensive dissection based on similar findings. One such example is Javid et. al. who reported a series of 241 lateral neck dissections for PTC and found level V involvement in 16.9% of cases [9]. Their series had a recurrence rate of 10.9%, all in patients who had comprehensive dissection of levels II–V, with 3 cases of recurrence involving level V. Again, these authors argue that level V dissection is always necessary as there is disease involvement in about one-fifth of cases.

There are several limitations to consider though when interpreting the results of the aforementioned studies. Primarily, these studies do not specify whether the positive nodes found in level V are macroscopic or microscopic disease, the latter of which may have less impact on clinically significant outcomes [10, 11]. Secondly, the method of marking neck levels in the specimen is not always reported, and for the studies that do specify this, there are a range of different methods used which confound the results. As such, we sought to determine if a selective approach to neck management in thyroid cancer with cervical metastases is associated with high recurrence rates, particularly in those undergoing less than comprehensive neck dissection of level Vb. Of the 204 neck dissections in our series, the overall regional control rate was high at 92%. With the selective dissection group, the 5-year regional control rate was equally high at 91%, with only 5 cases of regional recurrence in level V. The incidence of regional neck recurrence was the same regardless of whether comprehensive level V dissection was performed (8% vs 9% at 5 years, *p* = 0.89). The only statistically significant predictors for lateral neck recurrence on univariate analysis were total number of suspicious nodes on preoperative imaging, largest positive node on initial neck dissection, and whether patients received EBRT. On multivariate analysis, there remained no statistically significant difference in recurrence between the groups after accounting for the clinicopathologic variables associated with recurrence between the cohorts.

Patients were not randomized in our study to the type of neck dissection performed, which may represent a significant confounding factor. Although neck disease in level Vb on radiographic imaging is a clear indication for comprehensive level Vb dissection, the decision to perform this type of neck dissection at our center is not solely based on this finding alone. Other factors that guide the extent of neck dissection include the volume of disease (i.e. number of nodes and size of nodes), as well as location of the positive nodes outside of level Vb. This explains why 81% of the comprehensive cases in our series did not have level V disease on pre-operative imaging, and why the comprehensive group had larger pathological nodes on preoperative imaging (2.4 cm vs. 1.6 cm, *p* < 0.01), more total nodes on imaging (3.6 vs. 2.6, *p* = 0.03), and more positive nodes on the pathology specimen (6.7 vs. 5.2, *p* < 0.05). There were 4 patients who received selective neck dissection only but also had level V involvement on pre-operative imaging, which did not fit with our treatment philosophy. Two of these patients developed recurrence in the lateral neck. Since we reviewed the imaging retrospectively, it is possible that the level V involvement was originally missed at the time of surgery. The surgeons at the time may also have elected to perform an incomplete rather than comprehensive level V dissection with the plan to resect the nodal metastases from an anterior approach. Due to the retrospective nature of our study, we were also unable to determine TNM stage and histologic variants of PTC on all the patients, as some patients were referred for management of their neck with prior thyroidectomy performed at an outside center.

In our series, level V failure in the selective dissection patients only occurred in 5 out of 110 dissections (4.5%). This rate is lower than would be expected based on the literature, and there are several potential explanations for this difference. Firstly, some patients in our selective cohort may have benefited from a partial level V dissection, as the surgeons in this series frequently remove nodes in the anterior portion of level V during a selective neck dissection. Much of level Vb can be approached through an anterior approach as part of a “selective neck dissection”. Secondly, almost all patients in our study received adjuvant RAI as recommended in the ATA guidelines for all patients with clinical neck metastases (intermediate risk), which may reduce the rate of lateral neck recurrence in cases of microscopic nodal disease. In our series, we did not record whether the nodal metastases were microscopic or macroscopic. Micrometastatic disease may not significantly impact regional recurrence rates, independent of RAI use [10, 11].

Comprehensive level Vb neck dissection may place the spinal accessory nerve at greater risk of injury, as surgeons must dissect it from the nodal tissue of the posterior neck up to its entry into the anterior border of trapezius. Temporary or permanent injury to the nerve can occur from traction, devascularization or microtrauma and will result in shoulder-related disability characterized by shoulder droop, winged scapula, inability to shrug and dull non-localizing pain exacerbated by shoulder movement [12, 13]. We were not able to collect any data regarding shoulder morbidity in our study. However, we know from the existing literature that significant shoulder disability after comprehensive neck dissection including levels IIb and V will occur in up to 40% of patients, although much of this may be temporary [6]. Selective neck dissection, on the other hand, is associated with minimal shoulder morbidity, with patients exhibiting less shoulder impairment and fewer activity limitations when compared to comprehensive or radical neck dissections [6]. Additionally, extensive supraclavicular nodal dissection may result in increased rates of chylous fistula or seroma, and may put the brachial plexus and phrenic nerves at greater risk. If formal level Vb dissection does not improve regional recurrence rates, the surgeon would potentially avoid these additional morbidities by performing a selective, rather than comprehensive, lateral neck dissection.

## Conclusion

For PTC patients presenting with lateral neck metastases, a comprehensive level V dissection did not appear to reduce the rate of lateral neck recurrence over time. A selective neck dissection strategy to remove only the levels with suspicious nodes on pre-operative CT appeared to be equally effective. Selection bias within this study limits our ability to draw definitive conclusions regarding differences in regional recurrence between selective and comprehensive neck dissection.

## Abbreviations

CT: Computerized tomography
EBRT: External beam radiotherapy
ECE: Extracapsular extension
PTC: Papillary thyroid cancer
RAI: Radioactive iodine
